# Biodegradation of HA and β-TCP Ceramics Regulated by T-Cells

**DOI:** 10.3390/pharmaceutics14091962

**Published:** 2022-09-16

**Authors:** Zifan Zhao, Jing Zhang, Zaibo Yang, Qin Zhao

**Affiliations:** 1 The State Key Laboratory Breeding Base of Basic Science of Stomatology (Hubei-MOST) & Key Laboratory of Oral Biomedicine, Ministry of Education, School & Hospital of Stomatology, Wuhan University, Wuhan 430079, China; 2 Department of Stomatology, The Central Hospital of Enshi Tujia and Miao Autonomous Prefecture, Enshi 445000, China

**Keywords:** bioceramics, biodegradation, FBGCs, HA, immune response, T-cells, β-TCP

## Abstract

Biodegradability is one of the most important properties of implantable bone biomaterials, which is directly related to material bioactivity and the osteogenic effect. How foreign body giant cells (FBGC) involved in the biodegradation of bone biomaterials are regulated by the immune system is poorly understood. Hence, this study found that β-tricalcium phosphate (β-TCP) induced more FBGCs formation in the microenvironment (*p* = 0.0061) accompanied by more TNFα (*p* = 0.0014), IFNγ (*p* = 0.0024), and T-cells (*p* = 0.0029) than hydroxyapatite (HA), resulting in better biodegradability. The final use of T-cell depletion in mice confirmed that T-cell-mediated immune responses play a decisive role in the formation of FBGCs and promote bioceramic biodegradation. This study reveals the biological mechanism of in vivo biodegradation of implantable bone tissue engineering materials from the perspective of material-immune system interaction, which complements the mechanism of T-cells’ adaptive immunity in bone immune regulation and can be used as a theoretical basis for rational optimization of implantable material properties.

## 1. Introduction

The repair of large bone defects caused by trauma, tumors, inflammation, etc., is a common challenge for clinicians. Currently, bone tissue engineering uses bone replacement materials to repair bone defects, which not only avoids the secondary trauma caused by taking autologous bone, but also prepares personalized scaffolds to reconstruct the function and shape of bone according to the size of the defect. Thus, the use of bone replacement materials has become the best treatment strategy for bone defect repairs [1,2,3]. During the progress of bone tissue engineering, bioceramic scaffolds with calcium phosphate composition as the main component have received wide attention from researchers due to their chemical composition being similar to a bone’s chemical composition and having an excellent bone regeneration effect [4]. Degradability is often considered to be an important influence on the bioactivity of ceramics and is directly related to their regenerative effect in modulating bone defect repairs [5]. Therefore, researchers have worked to develop bioceramic scaffolds with superior degradability as a way to improve the bioactivity of bioceramic scaffolds. By using in situ polymerization techniques, p-HEMA was compounded on calcium phosphate ceramics to enhance their degradability without affecting biocompatibility [6]. In addition, the incorporation of hydroxyapatite into PLLA bone regeneration scaffolds was also claimed by the authors to take advantage of its improved degradation properties to enhance the bioactivity of the scaffold material [7]. However, these studies for improved degradability of ceramics were only optimized for the material’s basal degradation rate in solution and validated in cytological experiments, ignoring the more important material-host interaction, i.e., the complex microenvironment within the host of the biodegradation process of the material.

The implantation of biomaterials into the host is accompanied by a series of regeneration-related immune response events, including injury, tissue fluid-material interaction, temporary matrix formation, acute inflammation, chronic inflammation, granulation tissue development, foreign body reaction, and fibrosis [8,9]. The acute inflammatory response caused by biomaterial implantation usually subsides quickly within a week, followed by a foreign body response in which the immune system recognizes the biomaterial as foreign, during which mononuclear macrophages and their fused FBGCs attach to the material surface, absorbing and phagocytosing the ceramic and breaking it down. This is more conducive to the ion release and porous structure formation of the material and the formation of a naturally derived, biodegradable slow-release system around the material, which is essential to initiate the subsequent regeneration phase [10,11]. Overall, the basal degradation of the material and the biodegradation after implantation into the organism together determine the final degradation of the biomaterial in the complex environment of the organism, and the biodegradation is undoubtedly the more critical and closer to the actual one in terms of the impact on the regenerative effect of the material.

As the most direct participant in material biodegradation, FBGCs have an antigenic phenotype similar to that of mononuclear macrophages, which is strong evidence to confirm the formation of fusion of mononuclear-derived macrophages from FBGCs [12]. The osteoclast marker antitartrate acid phosphatase (TRAP) has been detected on FBGCs around implants and has become an important marker for the identification of FBGCs [13,14,15]. The formation of FBGCs is regulated by a variety of cell-matrix interactions and cytokines that recruit macrophages around the material and induce their fusion into FBGCs that participate in the biodegradation process of the implanted material [16,17]. The traditional view is that IL-4 and IL-13 are potent inducers of the fusion of peri-material macrophages into FBGCs [18,19]. After early acute inflammation subsides, a relatively stable microenvironment conducive to tissue regeneration develops around the material, during which lymphocytes appear around the material and dominate the adaptive immune response. Numerous studies have implied that lymphocytes may have a relatively long-lasting role in influencing macrophage fusion into FBGCs during the material tissue regeneration period [20,21,22], but whether lymphocytes have a decisive role in material biodegradation and their mechanism of action is not clear.

Although the main characteristics (density, porosity, pore volume, etc.) have a large influence on in vitro degradability, this study focuses on in vivo degradability, which is regulated by the immune microenvironment [23]. By comparing the immune microenvironment of HA and β-TCP degradation in vivo, this study speculated that the T-cell immune response closely related to FBGC might have an impact on the biodegradation process. Here, the present study focuses on the role of T-cell-mediated immune response in regulating FBGCs formation and its effect on the biodegradation properties of the material. This study found that there was a significant positive correlation between the number of T-cells and their associated inflammatory factors, TNF-α and IFN-γ, and the biodegradation properties of calcium phosphate ceramics. Moreover, the formation and biodegradation of FBGCs around calcium phosphate ceramics were significantly affected in T-cell-depleted mice. This study reveals that T-cell immune response may be an important factor affecting the biodegradation and biological effects of the material, enriching and complementing the fate process of biomaterials after implantation and providing the need for a more rational and accurate basis when improving and evaluating implantable materials.

## 2. Materials and Methods

### 2.1. Ethical Approval

The mice used in this study were handled in accordance with the policies of the Animal Research Ethics Committee of Wuhan University, Wuhan, China. The study protocol was approved by the Animal Use Ethics Committee of the Institute of Biomedical Sciences (protocol number S0792203014, A31/2020).

### 2.2. Preparation and Characterization of HA and β-TCP 

HA ceramics were prepared from HA powder according to the wet chemical method. After molding and polymerization in a mold, drying, pyrolysis, and finally sintering in air at 1250 °C for 8 h. β-TCP was prepared by sintering at 1200 °C for 8 h according to the method of previous studies [24,25]. The ideal chemical formula for β-TCP ceramics is Ca_3_(PO_4_)_2_ with a calcium to phosphorus ratio of 1.5. HA ceramics with the ideal chemical formula Ca_5_(PO_4_)_3_OH and a calcium to phosphorus ratio of 1.67 were used. X-ray diffraction (XRD) (Supplementary Figure S1A), Fourier transform infrared spectroscopy (FTIR) (Supplementary Figure S1B), and scanning electron microscopy (SEM) micrographs (Supplementary Figure S1C), were used to detect characterization of β-TCP and HA.

### 2.3. Implantation of Bioceramic Materials 

Eight-week-old female C57BL/6 mice were selected for animal experiments. Implantation was performed by making an incision in the skin and muscle with a scalpel after preoperative hair removal. The incision is approximately 8 mm long and precise to avoid damage to blood vessels. A material pellet of equal size is inserted into the wound with ophthalmic forceps and sutured to the muscle layer and skin.

### 2.4. Histological Staining

Muscle tissue-containing material was harvested and fixed in 4% paraformaldehyde for 24 h, followed by immersion in a frequently changed decalcifying solution containing 10% EDTA (ethylenediaminetetraacetic acid) and paraffin embedding by gradient alcohol immersion dehydration after 4 weeks. Paraffin specimens were prepared into 5 μm of thickness sections for subsequent staining. H and E, TRAP, immunofluorescenc (IF), and immunohistochemical staining (IHC) were used according to the manufacturer’s protocols (MXB Biotechnologies, Fuzhou Maixin Biotech. Co., Ltd., Fuzhou, China). The primary antibodies of IF and IHC included the following: F4/80 (1:200; ABclonal A1256, Wuhan, China), TNFα (1:200; ABclonal A0277, Wuhan, China), IL-1β (1:200; ABclonal A11369, Wuhan, China), IFNγ (1:200; ABclonal A12450, Wuhan, China), and CD3 (1:800; CST 86603S, Boston, MA, USA).

### 2.5. T-Cell-Depleted Mice

The hybridoma cell lines GK1.5 and 2.43 were cultured in RPMI-1640 (Hyclone, Logan, UT, USA) medium supplemented with 10% fetal bovine serum (Gibco, CA, USA) in cell incubator at 37 °C and 5% CO_2_. After the cell density reached 80%, the cells were cultured for 24 h. The supernatant of the cell culture was collected by centrifugation. The supernatant was injected intraperitoneally every 3 days to remove T-cells of the mice.

### 2.6. Statistical Analysis

GraphPad Prism software8 (San Diego, California, USA) was used for data analysis. Two-way ANOVA and Student’s *t*-test were used to assess differences between the two groups. *p* < 0.05 was considered a statistically significant difference.

## 3. Results and Discussion

### 3.1. FBGC-Mediated Biodegradation Is More Pronounced in β-TCP Than That in HA

**Figure 1:**
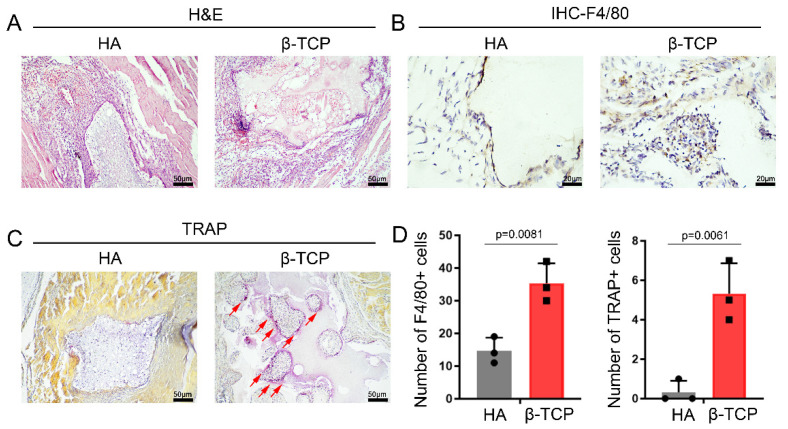
FBGC-mediated biodegradation of β-TCP is more pronounced than that of HA. (**A**) H and E staining showed the biodegradation of β-TCP and HA. (**B**) IHC staining of the macrophage’s marker F4/80. (**C**) TRAP staining showed the FBGCs marked by red arrows. (**D**) Semiquantitative comparison of positive expression. *n* = 3, black circles and blocks represent values from a single sample.

The preparation of HA and β-TCP is described in detail in Materials and Methods. To identify the possible impurity components of HA and β-TCP, XRD was used to analyze their elemental composition, and the characteristic peaks of β-TCP and HA were marked with arrows (Supplementary Figure S1A). FTIR spectra showed the absorption peaks of β-TCP and HA (Supplementary Figure S1B). SEM (Supplementary Figure S1C) revealed the porosity and pore volume of the material to some extent. HA and β-TCP were implanted in the mouse gastrocnemius muscle and taken for histological paraffin sectioning on day 14. H and E staining to observe the degradation of the materials showed that β-TCP presented a significant irregular morphology on day 14 in vivo, while HA maintained a relatively intact regular morphology (Figure 1A). This indicates that the overall degradation rate of β-TCP is higher than that of HA, but since there may be differences in the basal degradation rates of HA and β-TCP, we used immunohistochemical staining for the macrophage marker F4/80 and TRAP staining for macrophages and FBGCs around the material, respectively, to further observe the differences in the biodegradation of the two ceramics. The immunohistochemical results showed that the number of macrophages around β-TCP was significantly higher than that around HA. It also showed that a large number of macrophages were clustered in the absorption traps of β-TCP, while in HA, macrophages were mainly present on the surface of materials (Figure 1B,D). TRAP-positive cell expression was hardly detected around HA (Figure 1C,D). The above results suggest that the biodegradation rates of HA and β-TCP differ greatly after implantation and that macrophages and their aggregation to form FBGCs with phagocytosis are important reasons for the difference in biodegradation rates.

Whether FBGC-mediated biodegradation is beneficial or detrimental to the biological function of the material is still inconclusive. This question is primarily related to how the material performs its effects. For example, insulating materials in implantable pacemakers that are subject to biodegradation by FBGCs may cause device failure and a crisis in a patient’s life [26]. However, our study focused on bioceramic materials used for bone regeneration, where degradability is one of the most important properties that is closely related to the material’s effect on bone regeneration. It means that FBGCs and the biodegradation phenomenon directly determine the bone regeneration effect of bioceramic materials, which is the reason why most studies found β-TCP superior to HA [27,28,29,30]. 

The musculus gastrocnemius implantation model was used in this study because the immune microenvironment is homogeneous and free of interference from osteogenesis-related cells (skeletal stem cells, osteoblasts, osteoclasts, etc.) This environment is more suitable to study the interaction of immune cells with the material. Actually, it is important to study the role of scaffold materials on bone regeneration using a critical-size bone defect. However, while the present study focuses on the interaction of the material with immune cells, the critical bone defect model is more importantly concerned with the role of the material with bone-associated cells, and the two have a tendency to serve different research purposes. Similarly, in previously reported studies, muscle tissue implant models were widely used to evaluate bioceramic mechanisms [31,32,33], while animal models of critical bone defects were often used to evaluate the regenerative effects of bioceramics [34,35].

### 3.2. More Inflammatory Factors TNF-α and IFN-γ Surrounding β-TCP Than HA Induce Formation of FBGCs

**Figure 2:**
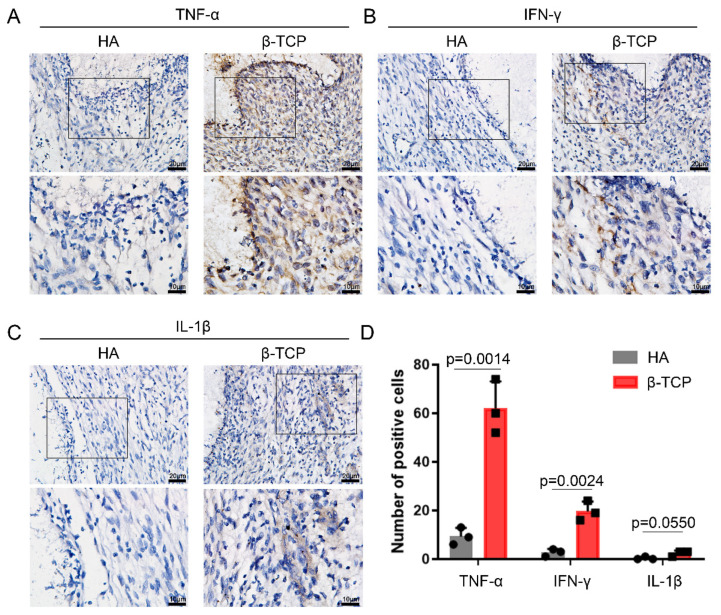
More inflammatory factors TNF-α and IFN-γ surrounding β-TCP than HA. IHC staining of (**A**) TNF-α, (**B**) IFN-γ, and (**C**) IL-1β surrounding β-TCP and HA. (**D**) Semiquantitative comparison of positive expression. *n* = 3, black circles and blocks represent values from a single sample. The larger version of the black frame area in (**A**–**C**) were shown in the image below.

The fusion of macrophages adhering to the surface of biomaterials as FBGCFBGC requires a highly coordinated series of events [36]. It has been shown that surface roughness has a large impact on FBGC formation, and it is generally believed that greater surface roughness facilitates FBGC formation and has the effect of accelerating bone remodeling, which is one of the reasons for the faster osseointegration of rough-surface implants in clinical practice [37]. In addition, changes in the ionic composition may also affect FBGC formation, such as Sr, Zn, and Mg-doped bioceramics, resulting in fewer FBGCs or reduced uptake of the material by FBGCs [38,39,40], while the addition of high concentrations of silicon may upregulate FBGC activity [41]. HA and β-TCP are both calcium phosphate ceramic materials with relatively similar surface morphology and elemental species, so why are there large differences in their surrounding FBGCs? It has been suggested that inflammatory factors in the microenvironment surrounding the materials are important factors [16]. IL-4 and IL-13 are among the factors that have been shown to regulate FBGC formation, but in addition, there are several factors that have yet to be defined to regulate FBGC formation [42,43,44]. The mechanisms and functions of FBGC formation are similar to those of osteoclast formation, and it has been shown that inflammatory factors, such as TNF-α, IFN-γ, and IL-1β can affect osteoclast formation in the absence of RANK-RANKL interactions [45,46,47,48]. Similar findings were found in the context of implanted materials, where FBGCs around the arthroplasty material expressed various cytokine receptors, such as IL-1R, IL-2R, and TNFR [17]. We examined three typical inflammatory cytokines including TNF-α, IL-1β, and IFN-γ around the material and showed that TNF-α and IFN-γ were significantly higher in the microenvironment around β-TCP than HA (Figure 2A,B,D), but no significant difference was detected for IL-1β (Figure 2C,D). This result suggests that inflammatory factors, such as TNF-α and IFN-γ may be critical in inducing macrophages to fuse into FBGCs and achieve material biodegradation.

### 3.3. More CD3+ T-Cells Surrounding β-TCP Than HA

**Figure 3:**
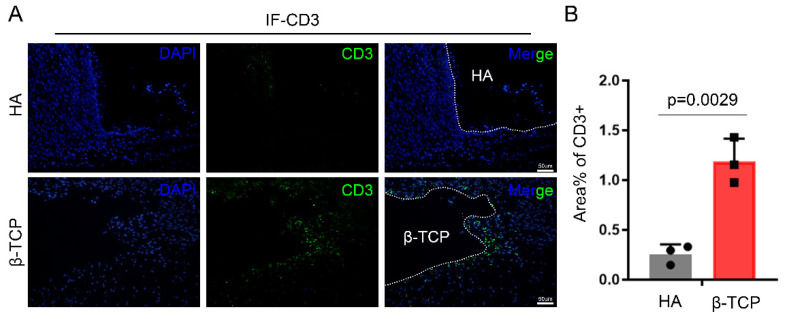
More CD3+ T-cells surrounding β-TCP than HA. (**A**) IHC staining of T-cells marker CD3. (**B**) Semiquantitative comparison of positive expression. *n* = 3, black circles and blocks represent values from a single sample.

A variety of immune cells are involved in the secretion of inflammatory factors. Resting immune cells can be activated in response to micro-organisms and their products, immune complexes, chemical mediators, and T lymphocyte-derived cytokines, which initiate the secretion of a large number of inflammatory mediators [12,49]. In addition, our results from a preliminary study of bioceramic material implantation showed that there was a burst of aggregation of natural immune cells, such as neutrophils, dendritic cells, and macrophages, for days 1–4 after the implantation procedure, followed by a substantial decrease and stabilization, with a peak of mainly T-lymphocytes at day 14 after the procedure [50]. We used immunofluorescence to stain T-cell marker CD3 around HA and β-TCP, and the results showed that the number of CD3+ T-cells was significantly higher around β-TCP than HA (Figure 3A,B). Thus, T-cells may be an important cause of higher inflammatory factors in the microenvironment around the material, which triggers the formation of FBGCs, leading to material biodegradation. Existing studies have shown that the coculture of T-cells with macrophages can promote macrophage fusion into FBGCs, but it is generally believed that T-cells are not a decisive factor for FBGC formation [22,51].

### 3.4. T-Cell Depletion Interferes with the FBGC-Mediated Biodegradation of β-TCP

**Figure 4:**
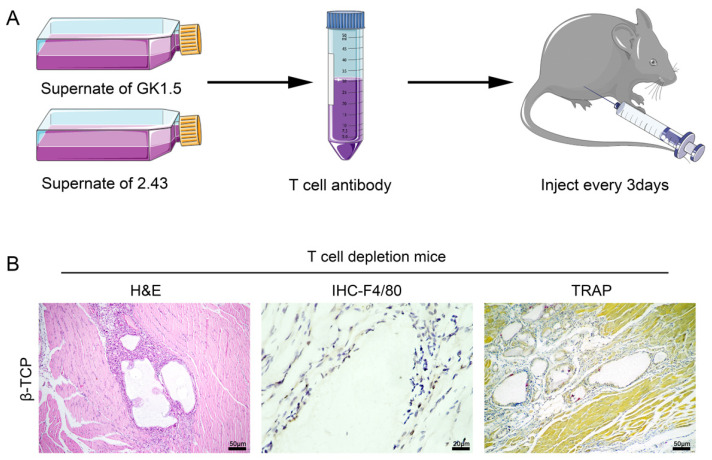
T-cell depletion interferes with the FBGC-mediated biodegradation of β-TCP. (**A**) Schematic diagram of T-cell depletion in an animal model. (**B**) H and E, IHC-F4/80, and TRAP staining showed the FBGC-mediated biodegradation of β-TCP in T-cell depletion in mice.

To confirm whether T-cell formation in FBGCs is necessary for formation in a bioceramic material implantation microenvironment, we cleared CD3+ T-cells in mice by intraperitoneal injection of hybridoma cells GK1.5 and 2.43 culture supernatants (Figure 4A). Although the nude mouse has been used as a conventional animal model for T-cell development defects, congenital systemic T-cell development defects have been reported to have significant effects on the hematopoietic system and metabolism, which are important factors affecting bone repair in addition to adaptive immune responses [52,53]. Therefore, we chose to use depleting antibodies to clear T-cells to obtain the least disruptive effect. Therefore, we injected antibodies every 3 days to ensure that the clearance efficiency of T-cells was maintained at 90% during the model study phase. This method has been accepted by the industry and has been used in several studies, meeting experimental standards [50,54]. The T-cell depletion mice were implanted with β-TCP in the gastrocnemius muscle, and paraffin sections were taken 14 days later, and HE staining showed that β-TCP remained relatively intact in the T-cell depletion mice with a reduced degradation rate. Immunohistochemistry and TRAP staining showed that F4/80+ macrophages were present less around the material and almost no FBGC formation was detected (Figure 4B). The above results confirm that the absence of T-cells present directly leads to the inability of FBGCs to form and affects the biodegradation of the material.

Ultimately, this study concluded that T-cells surrounding β-TCP play a role in inducing macrophages to form FBGCs in the calcium phosphate ceramic implantation microenvironment, similar to previous studies with other materials. The difference, however, is that our study shows that T-cells play a decisive rather than a secondary role in the biodegradation of calcium phosphate ceramics [51,55]. However, the assay method in this study is not perfect, and it is unclear whether this conclusion is applicable to other types of materials, which will be further added in the subsequent study. It is crucial to the further understanding of the biodegradation process and the generation of biological effects of calcium phosphate materials.

## 4. Conclusions

In this study, we found that β-TCP achieved better biodegradability than HA by activating T-cell-mediated immune responses and inducing macrophage fusion into more FBGCs (*p* = 0.0061). The conclusion of this study was the different biodegradation effects of using the same β-TCP material with identical properties in the presence or absence of T-cells. The present study is instructive for the improvement of biomaterial properties. It is advocated that immunological properties of implantable materials should not be improved exclusively by the suppression of inflammatory response and immune cell activity, likewise, the regenerative properties should not be improved exclusively for the promotion of osteoblast differentiation and inhibition of osteoclastic differentiation. The appropriate immune response and osteoclast activity are necessary for a desirable bone remodeling environment involving biomaterials.

## Data Availability

Not applicable.

## Supplementary Materials

The following supporting information can be downloaded at: https://www.mdpi.com/article/10.3390/pharmaceutics14091962/s1, Figure S1: Characterization of β-TCP and HA.

## References

[1] Rodriguez R.U., Kemper N., Breathwaite E., Dutta S.M., Huber A., Murchison A., Chen S., Hsu E.L., Hsu W.K., Francis M.P. (2016). Demineralized bone matrix fibers formable as general and custom 3D printed mold-based implants for promoting bone regeneration. Biofabrication.

[2] Chu Y.S., Wong P.-C., Jang J.S.-C., Chen C.-H., Wu S.-H. (2022). Combining Mg–Zn–Ca Bulk Metallic Glass with a Mesoporous Silica Nanocomposite for Bone Tissue Engineering. Pharmaceutics.

[3] Jiang Q., Bai G., Liu X., Chen Y., Xu G., Yang C., Zhang Z. (2021). 3D GelMA ICC Scaffolds Combined with SW033291 for Bone Regeneration by Modulating Macrophage Polarization. Pharmaceutics.

[4] Bouler J., Pilet P., Gauthier O., Verron E. (2017). Biphasic calcium phosphate ceramics for bone reconstruction: A review of biological response. Acta Biomater..

[5] Su J., Hua S., Chen A., Chen P., Yang L., Yuan X., Qi D., Zhu H., Yan C., Xiao J. (2021). Three-dimensional printing of gyroid-structured composite bioceramic scaffolds with tuneable degradability. Biomater. Adv..

[6] Huang J., Ten E., Liu G., Finzen M., Yu W., Lee J.S., Saiz E., Tomsia A.P. (2013). Biocomposites of pHEMA with HA/β-TCP (60/40) for bone tissue engineering: Swelling, hydrolytic degradation, and in vitro behavior. Polymer.

[7] Shuai C., Yang W., Feng P., Peng S., Pan H. (2020). Accelerated degradation of HAP/PLLA bone scaffold by PGA blending facilitates bioactivity and osteoconductivity. Bioact. Mater..

[8] Anderson J.M. (2000). Multinucleated giant cells. Curr. Opin. Hematol..

[9] Luttikhuizen D.T., Harmsen M.C., Van Luyn M.J. (2006). Cellular and Molecular Dynamics in the Foreign Body Reaction. Tissue Eng..

[10] Gretzer C., Emanuelsson L., Liljensten E., Thomsen P. (2006). The inflammatory cell influx and cytokines changes during transition from acute inflammation to fibrous repair around implanted materials. J. Biomater. Sci. Polym. Ed..

[11] Zhang Q., Xiao L., Xiao Y. (2021). Porous Nanomaterials Targeting Autophagy in Bone Regeneration. Pharmaceutics.

[12] Anderson J.M., Rodriguez A., Chang D.T. (2008). Foreign body reaction to biomaterials. Semin. Immunol..

[13] Quinn J.M.W., Athanasou N.A., McGee J. (1991). Extracellular matrix receptor and platelet antigens on osteoclasts and foreign body giant cells. Histochem. Cell Biol..

[14] Kadoya Y., Al-Saffar N., Kobayashi A., Revell P. (1994). The expression of osteoclast markers on foreign body giant cells. Bone Miner..

[15] Klopfleisch R. (2016). Macrophage reaction against biomaterials in the mouse model–Phenotypes, functions and markers. Acta Biomater..

[16] Athanasou N.A., Quinn J. (1990). Immunophenotypic differences between osteoclasts and macrophage polykaryons: Immunohistological distinction and implications for osteoclast ontogeny and function. J. Clin. Pathol..

[17] Neale S.D., Athanasou N.A. (1999). Cytokine receptor profile of arthroplasty macrophages, foreign body giant cells and mature osteoclasts. Acta Orthop. Scand..

[18] McNally A.K., Anderson J.M. (1995). Interleukin-4 induces foreign body giant cells from human monocytes/macrophages. Differential lymphokine regulation of macrophage fusion leads to morphological variants of multinucleated giant cells. Am. J. Pathol..

[19] DeFife K.M., Jenney C.R., McNally A.K., Colton E., Anderson J.M. (1997). Interleukin-13 induces human monocyte/macrophage fusion and macrophage mannose receptor expression. J. Immunol..

[20] Trout K., Holian A. (2020). Multinucleated giant cell phenotype in response to stimulation. Immunobiology.

[21] Trindade M. (2001). In vitro reaction to orthopaedic biomaterials by macrophages and lymphocytes isolated from patients undergoing revision surgery. Biomaterials.

[22] Brodbeck W.G., MacEwan M., Colton E., Meyerson H., Anderson J.M. (2005). Lymphocytes and the foreign body response: Lymphocyte enhancement of macrophage adhesion and fusion. J. Biomed. Mater. Res. Part A.

[23] Stastny P., Sedlacek R., Suchy T., Lukasova V., Rampichova M., Trunec M. (2019). Structure degradation and strength changes of sintered calcium phosphate bone scaffolds with different phase structures during simulated biodegradation in vitro. Mater. Sci. Eng. C.

[24] Wu C., Chang J., Zhai W., Ni S. (2007). A novel bioactive porous bredigite (Ca_7_MgSi_4_O_16_) scaffold with biomimetic apatite layer for bone tissue engineering. J. Mater. Sci. Mater. Med..

[25] Liao L., Yang S., Miron R.J., Wei J., Zhang Y., Zhang M. (2014). Osteogenic Properties of PBLG-g-HA/PLLA Nanocomposites. PLoS ONE.

[26] Wiggins M.J., Wilkoff B., Anderson J.M., Hiltner A. (2001). Biodegradation of polyether polyurethane inner insulation in bipolar pacemaker leads. J. Biomed. Mater. Res..

[27] Yamada S. (1997). Osteoclastic resorption of calcium phosphate ceramics with different hydroxyapatite/β-tricalcium phosphate ratios. Biomaterials.

[28] Lodoso-Torrecilla I., Beucken J.V.D., Jansen J. (2020). Calcium phosphate cements: Optimization toward biodegradability. Acta Biomater..

[29] Tew M., Damstra-Wijmenga S. (1991). Safest birth attendants: Recent Dutch evidence. Midwifery.

[30] Matos S., Guerra F., Krauser J.T., Figueiredo H., Marcelino J.P., Sanz M. (2011). Evaluation of an anorganic bovine-derived mineral with P-15 hydrogel bone graft: Preliminary study in a rabbit cranial bone model. Clin. Oral Implant. Res..

[31] Cheng L., Khalaf A.T., Lin T., Ran L., Shi Z., Wan J., Zhou X., Zou L. (2020). Exercise Promotes the Osteoinduction of HA/β-TCP Biomaterials via the Wnt Signaling Pathway. Metabolites.

[32] Cheng L., Liu Z., Yan S., Chen Z., Zou L., Shi Z. (2019). The role of osteoclasts in osteoinduction triggered by calcium phosphate biomaterials in mice. Bio-Med. Mater. Eng..

[33] Fellah B.H., Josselin N., Chappard D., Weiss P., Layrolle P. (2007). Inflammatory reaction in rats muscle after implantation of biphasic calcium phosphate micro particles. J. Mater. Sci. Mater. Electron..

[34] Johnson Z.M., Yuan Y., Li X., Jashashvili T., Jamieson M., Urata M., Chen Y., Chai Y. (2021). Mesenchymal Stem Cells and Three-Dimensional-Osteoconductive Scaffold Regenerate Calvarial Bone in Critical Size Defects in Swine. Stem Cells Transl. Med..

[35] Susin C., Lee J., Fiorini T., Koo K.-T., Schupbach P., Angst P.D.M., Stadler A.F., Wikesjö U.M. (2018). Screening of candidate biomaterials for alveolar augmentation using a critical-size rat calvaria defect model. J. Clin. Periodontol..

[36] Chen E.H., Grote E., Mohler W., Vignery A. (2007). Cell-cell fusion. FEBS Lett..

[37] Makihira S., Mine Y., Kosaka E., Nikawa H. (2007). Titanium Surface Roughness Accelerates RANKL-dependent Differentiation in the Osteoclast Precursor Cell Line, RAW264.7. Dent. Mater. J..

[38] Gentleman E., Fredholm Y.C., Jell G., Lotfibakhshaiesh N., O’Donnell M.D., Hill R.G., Stevens M.M. (2010). The effects of strontium-substituted bioactive glasses on osteoblasts and osteoclasts in vitro. Biomaterials.

[39] Roy M., Fielding G.A., Bandyopadhyay A., Bose S. (2012). Effects of zinc and strontium substitution in tricalcium phosphate on osteoclast differentiation and resorption. Biomater. Sci..

[40] Roy M., Bose S. (2012). Osteoclastogenesis and osteoclastic resorption of tricalcium phosphate: Effect of strontium and magnesium doping. J. Biomed. Mater. Res. Part A.

[41] Vahabzadeh S., Roy M., Bose S. (2015). Effects of silicon on osteoclast cell mediated degradation, in vivo osteogenesis and vasculogenesis of brushite cement. J. Mater. Chem. B.

[42] McNally A.K., Anderson J.M. (2014). Phenotypic expression in human monocyte-derived interleukin-4-induced foreign body giant cells and macrophages in vitro: Dependence on material surface properties. J. Biomed. Mater. Res. Part A.

[43] Saleh L.S., Vanderheyden C., Frederickson A., Bryant S.J. (2020). Prostaglandin E2 and Its Receptor EP2 Modulate Macrophage Activation and Fusion In Vitro. ACS Biomater. Sci. Eng..

[44] Collier T.O., Anderson J.M. (2002). Protein and surface effects on monocyte and macrophage adhesion, maturation, and survival. J. Biomed. Mater. Res..

[45] Azuma Y., Kaji K., Katogi R., Takeshita S., Kudo A. (2000). Tumor Necrosis Factor-α Induces Differentiation of and Bone Resorption by Osteoclasts. J. Biol. Chem..

[46] Lamichhane S., Anderson J.A., Vierhout T., Remund T., Sun H., Kelly P. (2017). Polytetrafluoroethylene topographies determine the adhesion, activation, and foreign body giant cell formation of macrophages. J. Biomed. Mater. Res. Part A.

[47] Ainslie K.M., Bachelder E.M., Borkar S., Zahr A.S., Sen A., Badding J.V., Pishko M.V. (2006). Cell Adhesion on Nanofibrous Polytetrafluoroethylene (nPTFE). Langmuir.

[48] Khandwekar A., Rho C.K. (2012). Modulation of cellular responses on engineered polyurethane implants. J. Biomed. Mater. Res. Part A.

[49] Fujiwara N., Kobayashi K. (2005). Macrophages in Inflammation. Curr. Drug Target -Inflamm. Allergy.

[50] Zhao Z., Zhao Q., Gu B., Yin C., Shen K., Tang H., Xia H., Zhang X., Zhao Y., Yang X. (2020). Minimally invasive implantation and decreased inflammation reduce osteoinduction of biomaterial. Theranostics.

[51] Rodriguez A., MacEwan S.R., Meyerson H., Kirk J.T., Anderson J.M. (2008). The foreign body reaction in T-cell-deficient mice. J. Biomed. Mater. Res. Part A.

[52] Iriguchi S., Kikuchi N., Kaneko S., Noguchi E., Morishima Y., Matsuyama M., Yoh K., Takahashi S., Nakauchi H., Ishii Y. (2015). T-cell–restricted T-bet overexpression induces aberrant hematopoiesis of myeloid cells and impairs function of macrophages in the lung. Blood.

[53] Pearce E.L., Pearce E.J. (2013). Metabolic Pathways in Immune Cell Activation and Quiescence. Immunity.

[54] Kishton R.J., Sukumar M., Restifo N.P. (2017). Metabolic Regulation of T Cell Longevity and Function in Tumor Immunotherapy. Cell Metab..

[55] Yang J., Jao B., McNally A.K., Anderson J.M. (2014). In vivo quantitative and qualitative assessment of foreign body giant cell formation on biomaterials in mice deficient in natural killer lymphocyte subsets, mast cells, or the interleukin-4 receptorα and in severe combined immunodeficient mice. J. Biomed. Mater. Res. Part A.

