# Supplementary material for: Biodegradation of HA and β-TCP Ceramics Regulated by T-Cells
Source: Pharmaceutics. 2022 Sep 16;14(9):1962. doi: 10.3390/pharmaceutics14091962 (PMC9502083; doi:10.3390/pharmaceutics14091962)
Supplement: Supplementary file 1 [file pharmaceutics-14-01962-s001.zip › pharmaceutics-1821266-supplementary.pdf]

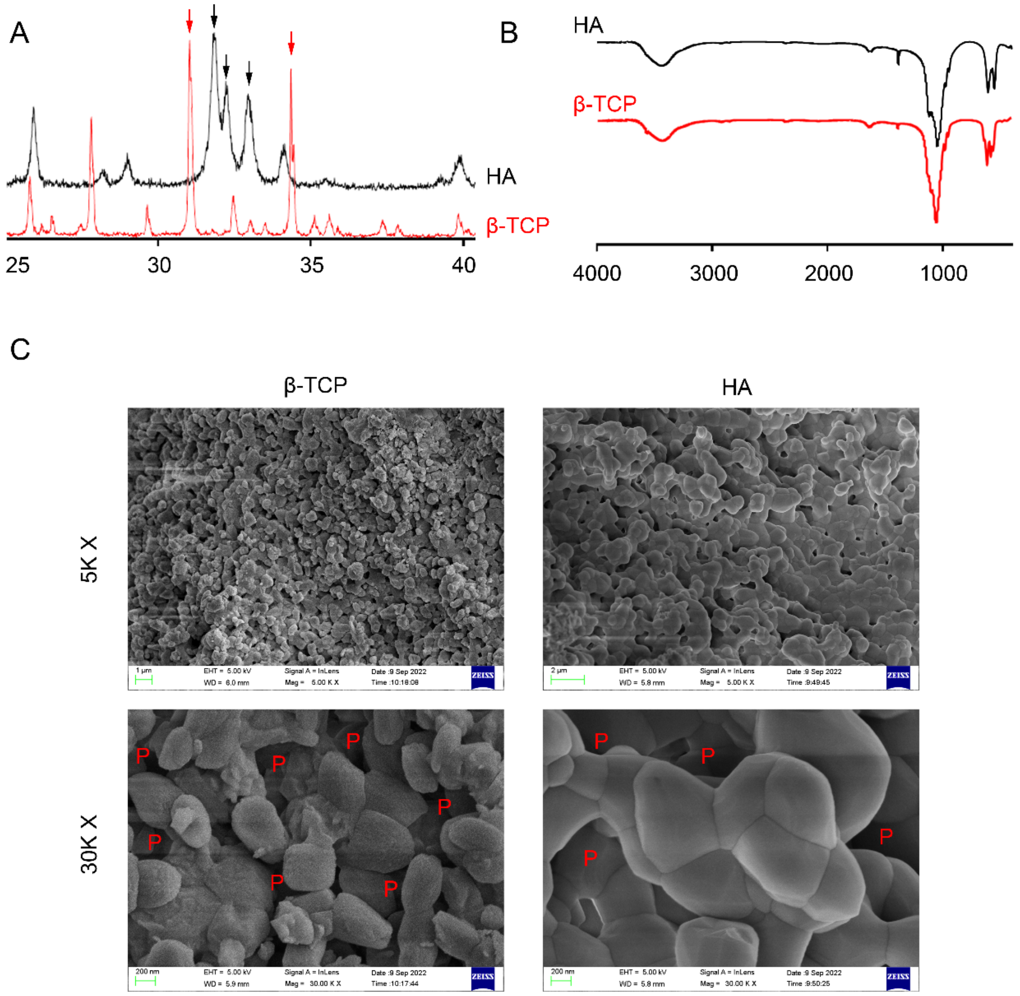

Supplementary Figure S1. Characterization of β-TCP and HA. (**A**) X-ray diffraction (XRD) of β-TCP (red) and HA (black). The characteristic peak marked by arrows. (**B**) Fourier transform infrared spectroscopy (FTIR) of β-TCP (red) and HA (black). (**C**) Scanning electron microscopy (SEM) of β-TCP and HA. “P” stands for pores.
